# Prevalence and types of psychological distress in breast cancer patients: An analysis of consultation delay

**DOI:** 10.1192/j.eurpsy.2025.1240

**Published:** 2025-08-26

**Authors:** H. Ktari, A. Hadj Salah, L. Marzouki, A. Daldoul, S. Zaied, B. Amamou

**Affiliations:** 1 Psychiatry; 2 Cardiology, Fattouma Bourguiba Hospital, Monastir, Tunisia

## Abstract

**Introduction:**

Breast cancer is the leading cancer among women, and its early diagnosis is vital for improving survival rates. However, many women delay seeking medical attention after noticing symptoms. Factors such as lack of awareness, fear of diagnosis, financial constraints, and family responsibilities often contribute to these delays. In addition to the physical burden of cancer, psychological distress is a common but often overlooked aspect of the patient experience.

**Objectives:**

To identify the prevalence and causes of delayed consultation as well as the different types of psychological distress (practical, familial, emotional, spiritual, and physical) among patients with breast cancer.

**Methods:**

We conducted a descriptive and analytical study over a one-year period (June 2021 - May 2022) in the oncology department of the Maternity and Neonatology Center of Monastir. Data were collected using information sheets, interviews, and medical records. Psychological impact was assessed using the Distress Thermometer (DT), a visual scale from 0 to 10, accompanied by a questionnaire on practical, familial, emotional, spiritual, and physical issues. The median consultation delay and reasons for the delay were also examined.

**Results:**

Among the 150 patients diagnosed with breast cancer who received treatment and follow-up at the Maternity and Neonatology Center of Monastir, the median consultation delay was 28 days, with delays ranging from 1 to 912 days. Thirty-five patients (23.3%) experienced late consultations, defined as a delay of more than 90 days. The main reasons for the delay included 62.2% of patients not suspecting cancer after the first functional signs, 11.1% fearing the diagnosis, and 8.9% facing financial issues or family and professional responsibilities. Among the 104 patients who completed the Distress Thermometer questionnaire, 31.7% had a distress level below 4, while 68.3% had a distress level of 4 or above. The most frequently reported practical problems were child care (26.92%) and meal preparation (18.26%). Familial problems mainly concerned relationships with children (26.92%). Emotional issues included fear (50%), nervousness (50.96%), and sadness (50%). Regarding spiritual concerns, 32.6% of patients had spiritual or religious concerns, and 67 patients reported physical problems.

**Conclusions:**

The results show that the majority of patients experience high psychological distress, particularly in emotional and practical domains. The median consultation delay is 28 days, with a significant delay in 23.3% of patients. It is therefore crucial to improve awareness and psychological support to reduce consultation delays and optimize breast cancer management and patient well-being.

**Disclosure of Interest:**

None Declared

